# Diagnosis of Microscopic Polyangiitis by EBUS-Guided Transbronchial Mediastinal Cryobiopsy: A Case Report

**DOI:** 10.3390/diagnostics16010125

**Published:** 2026-01-01

**Authors:** Miriam Retuerto-Guerrero, Javier Juan-García, Pablo Franco-Suárez, Samuel Saez-Álvarez, Octavio Miguel Rivero-Lezcano, Elvira Díez-Álvarez

**Affiliations:** 1 Rheumatology Department, Complejo Asistencial Universitario de León, 24008 León, Spain; elviraleon009@gmail.com; 2 Pulmonology Department, Interventional Pulmonology Unit, Complejo Asistencial Universitario de León, 24008 León, Spain; jjuang@saludcastillayleon.es (J.J.-G.); pfrancosu@saludcastillayleon.es (P.F.-S.); 3 Pathology Department, Complejo Asistencial Universitario de León, 24008 León, Spain; ssaeza@saludcastillayleon.es; 4 Research Unit, Complejo Asistencial Universitario de León, 24008 León, Spain; orivero@saludcastillayleon.es; 5 Institute of Biomedicine (IBIOMED), Universidad de León, 24004 León, Spain

**Keywords:** microscopic polyangiitis, EBUS-TMC, mediastinal lymphadenopathy, ANCA-associated vasculitis

## Abstract

**Background and Clinical Significance:** Isolated mediastinal lymphadenopathy is an exceptionally rare and diagnostically challenging initial manifestation of microscopic polyangiitis (MPA), often mimicking malignancy or infection. This case highlights the pivotal role of an innovative minimally invasive technique in achieving a definitive diagnosis. To the best of our knowledge, this is the first reported case of MPA diagnosed via EBUS-TMC. **Case Presentation:** A 55-year-old male livestock farmer from a rural area with a history of recurrent pneumonia presented with four weeks of persistent fever, significant weight loss (7 kg), myalgia, and asthenia. Physical examination revealed fever and cachexia. Notable findings included leukocytosis (17,000/μL), normocytic anemia, thrombocytosis (672,000/μL), highly elevated inflammatory markers (CRP 145 mg/L, ESR 120 mm/h), and strongly positive MPO-ANCA (>134 U/mL). Serological testing was significant for IgG antibodies against *Coxiella burnetii* (Phase I 1:64, Phase II 1:256). PET-CT imaging demonstrated hypermetabolic bilateral hilar and mediastinal lymphadenopathy. Diagnostic challenges included overlapping serological findings suggestive of past *Coxiella burnetii* exposure. Endobronchial ultrasound–guided transbronchial mediastinal cryobiopsy (EBUS-TMC) of a subcarinal lymph node was performed, providing a high-quality sample that revealed neutrophilic small-vessel vasculitis with fibrinoid necrosis, definitive for MPA. Immunosuppressive therapy with high-dose corticosteroids and rituximab (1000 mg on days 1 and 15) was initiated, leading to the complete resolution of all constitutional symptoms. **Conclusions:** This case illustrates that EBUS-TMC is a safe and highly effective diagnostic tool for obtaining critical histological evidence in systemic vasculitides with atypical presentations. This technique should be considered in the diagnostic algorithm for unexplained mediastinal lymphadenopathy to avoid more invasive surgical procedures.

## 1. Introduction

Microscopic polyangiitis (MPA) is a rare necrotizing vasculitis associated with anti-neutrophil cytoplasmic antibodies (ANCA) [1], typically targeting small vessels and often presenting with renal and pulmonary involvement. While pulmonary manifestations are common, isolated mediastinal lymphadenopathy as the primary presentation is exceptionally rare and poses a significant diagnostic challenge, often mimicking lymphoma, infection, or sarcoidosis [2]. Beyond its rarity, isolated mediastinal lymphadenopathy in suspected systemic vasculitis creates a specific diagnostic gap: the clinical probability often favors infection, granulomatous disease, or lymphoma, yet delays in obtaining architecture-preserving tissue can postpone definitive therapy. Conventional endobronchial ultrasound-guided transbronchial needle aspiration (EBUS-TBNA) remains the most efficient first step for mediastinal and hilar nodes; however, cytology-dominant samples may be suboptimal when the diagnostic signal resides in microanatomical relationships—such as vessel–wall injury, perivascular inflammation, or intact granulomatous patterns. In this niche, endobronchial ultrasound-guided transbronchial mediastinal cryobiopsy (EBUS-TMC) is designed to retrieve larger, minimally crushed cores that maintain histo-architectural integrity and support extended ancillary testing (immunohistochemistry, special stains, and—when relevant—molecular assays) [3].

Importantly, the clinical rationale for EBUS-TMC extends beyond neoplasia. In systemic vasculitis, histologic confirmation remains the gold standard when organ-directed biopsy is impractical or unsafe. Nodal tissue that preserves architecture can demonstrate small-vessel vasculitic injury—including neutrophilic infiltrates and fibrinoid necrosis—while simultaneously excluding key mimics such as lymphoma or sarcoidosis. In practice, this “architecture-first” strategy can shorten time-to-diagnosis, reduce the need for surgical procedures (e.g., mediastinoscopy), and expedite the initiation of immunosuppression in appropriate cases. Procedural safety has been favorable in contemporary series when standardized steps are followed (Doppler-guided avascular trajectories, avoidance of necrotic areas, controlled tract creation, and planned hemostasis), and the learning curve appears modest for teams experienced in EBUS [4].

This report therefore addresses a dual objective. First, it documents a unique presentation of MPA in which isolated mediastinal lymphadenopathy was the dominant finding and in which EBUS-TMC provided the decisive histopathological evidence for diagnosis. Second, it contextualizes the role of EBUS-TMC within a pragmatic diagnostic pathway: EBUS-TBNA remains the front-line approach for most mediastinal nodes, while early, same-session escalation to EBUS-TMC should be considered when preserved architecture is essential or when initial cytology is inconclusive. By highlighting technical feasibility, safety, and the specific diagnostic value of architecture-preserving cores in vasculitis, this case supports the careful incorporation of EBUS-TMC into multidisciplinary algorithms for unexplained mediastinal lymphadenopathy. To our knowledge, no prior report has established MPA from mediastinal lymph node tissue via EBUS-TMC, underscoring the novelty and specific contribution of our case.

## 2. Materials and Methods

Written informed consent for publication was obtained. Clinical, laboratory, and imaging data were abstracted from the electronic health record; the work-up included complete blood count, comprehensive metabolic panel, erythrocyte sedimentation rate/C-reactive protein (ESR/CRP), urine microscopy, and antineutrophil cytoplasmic antibodies (ANCA) by enzyme-linked immunosorbent assay (ELISA) targeting myeloperoxidase/proteinase 3 (MPO/PR3), plus a targeted microbiological assessment according to epidemiology to exclude infectious lymphadenopathy. Thoracic evaluation comprised contrast-enhanced chest computed tomography (CT) and positron emission tomography–computed tomography (PET–CT) to characterize mediastinal lymph nodes and rule out alternative foci. The mediastinal EBUS-TMC was performed under EBUS guidance with a linear EBUS bronchoscope and real-time ultrasound mapping; a flexible cryoprobe (1.1 mm) was advanced with 3–5 s freeze times to obtain 2–3 cores (≈5–8 mm). Immunohistochemistry was applied to exclude lymphoma, granulomatous disease, and metastasis, and small- to medium-vessel vasculitic changes were specifically assessed within nodal architecture. 

## 3. Case Presentation

A 55-year-old male livestock farmer from a rural area, active smoker (1 pack/day), with dyslipidemia and a history of recurrent pneumonia since youth (previously evaluated by Immunology, ruling out primary or secondary immunodeficiencies), presented with four weeks of persistent fever (up to 38 °C), significant weight loss (7 kg over two months), myalgia, and asthenia. Despite empirical antibiotic regimens including azithromycin and levofloxacin, his symptoms persisted. Due to positive Chlamydia pneumoniae serology (IgM/IgG+), doxycycline was initiated but failed to improve symptoms.

Laboratory results demonstrated leukocytosis (17,000/μL), normocytic anemia (hemoglobin 10.1 g/dL), thrombocytosis (672,000/μL), elevated inflammatory markers (CRP 145 mg/L, ESR 120 mm/h), and strongly positive perinuclear MPO-ANCA > 134 U/mL. Protein electrophoresis indicated an inflammatory profile. Creatine kinase levels were normal. Renal function showed serum creatinine level of 1.03 mg/dL and an estimated glomerular filtration rate of 81 mL/min/1.73 m^2^, with microhematuria and proteinuria (0.32 g/24 h). Nephrology did not indicate renal biopsy due to preserved function.

Serological testing revealed *Coxiella burnetii* immunoglobulin G Phase I (1:64) and Phase II (1:256) titers, while immunoglobulin M antibodies were negative. Other serologies were negative for hepatitis A virus, hepatitis B virus, hepatitis C virus, human immunodeficiency virus, syphilis, Brucella, and cytomegalovirus. Epstein–Barr virus, Borrelia, and Rickettsia showed immunoglobulin G positive but immunoglobulin M negative. Interferon-gamma release assay and Mantoux test were negative. Urine culture showed scant mixed Gram-positive flora, and blood cultures were negative. Echocardiography showed no evidence of endocarditis.

**Figure 1:**
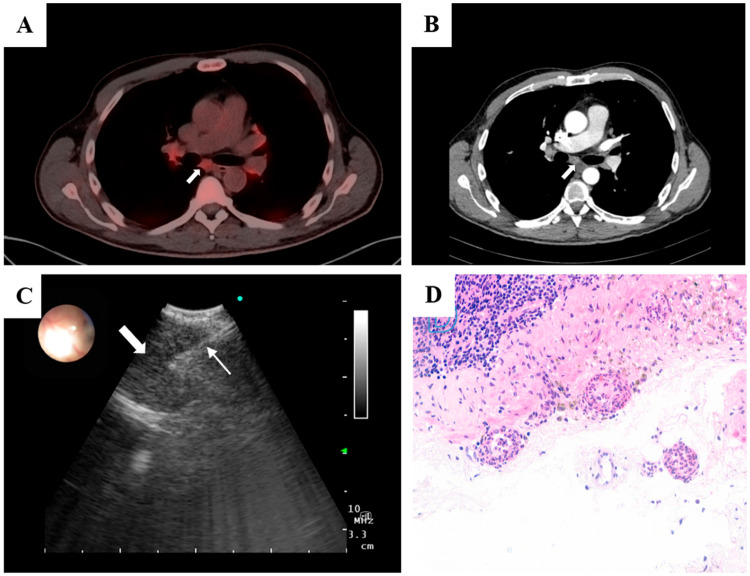
(**A**) PET–CT showing bilateral hypermetabolic hilar and mediastinal lymphadenopathy (SUV_max 4.0–5.0), including a 9 mm subcarinal node (SUV_max 4.6); no FDG-avid primary tumor or large-vessel uptake. (**B**) Axial contrast-enhanced CT depicting the subcarinal lymph node corresponding to the PET-avid focus. (**C**) Linear EBUS view after the tunneling approach showing the 1.1 mm cryoprobe (thin arrow) within the target subcarinal node (thick arrow), with real-time Doppler used to confirm an avascular trajectory. (**D**) High-power photomicrograph (H&E) of the nodal capsule demonstrating small-vessel vasculitis with leukocytoclasia, segmental vessel-wall destruction, and fibrinoid necrosis, with preserved nodal architecture and no granulomas or malignancy.

PET–CT showed hypermetabolic bilateral hilar and mediastinal lymphadenopathy (SUVmax 4.0–5.0) with incipient bilateral basilar pulmonary fibrosis. There was no evidence of malignancy or large-vessel vasculitis. Given the PET–CT pattern without an identifiable primary, negative blood and nodal cultures with a normal echocardiogram, and lack of response to appropriate antibiotics, infectious and malignant etiologies were deprioritized. Because architecture-preserving tissue was needed to assess vessel-wall injury and exclude granulomatous disease, we selected EBUS-TMC to obtain an intact core from the small subcarinal node. Histology demonstrated neutrophilic small-vessel vasculitis with fibrinoid necrosis; together with high-titer MPO-ANCA and clinical improvement only after immunosuppression, these findings supported MPA. EBUS (BF-UC180F, Olympus, Tokyo, Japan) identified a 9 mm subcarinal lymph node (maximum standardized uptake value 4.6). A transbronchial needle aspiration was performed using a 22G EBUS-TBNA needle (SonoTip® TopGain, Medi-Globe) and the Ariza-Pallarés tunneling method, followed by an EBUS-TMC of the node with a 1.1-mm cryoprobe (Erbecryo 20402-401, Tübingen, Germany), obtaining high-quality samples without complications (Figure 1). Histopathological examination confirmed neutrophilic small-vessel vasculitis with fibrinoid necrosis, consistent with microscopic polyangiitis. No granulomas or malignant cells were observed. Microbiological cultures of the sample showed no growth.

**Figure 2:**
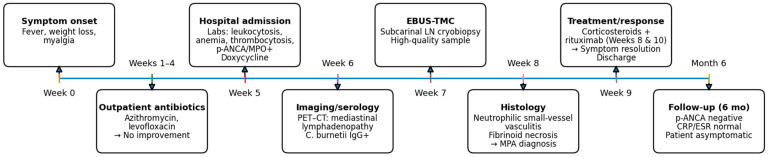
Overview of clinical course and key interventions (refer to Table 1 for details).

Following histopathological confirmation, immunosuppressive therapy was initiated with high-dose systemic steroid (1 mg/kg/day) and rituximab (1000 mg on days 1 and 15), achieving complete resolution of constitutional symptoms. A chronological summary of the diagnostic workup, interventions, and outcomes is provided in Table 1 (detailed) and Figure 2 (graphical overview).

## 4. Discussion

MPA is a necrotizing vasculitis of small- to medium-sized vessels, with an annual incidence estimated at 1.5–16 per million person-years and a prevalence of 39.2 (35.8–42.7) per million persons [5,6]. The disease typically manifests with a prolonged prodrome of constitutional symptoms including fever, weight loss, and malaise, eventually progressing to overt organ involvement. Classic presentations include renal disease (80–100% of cases), pulmonary manifestations (25–80%), cutaneous involvement (20–70%), and neurological symptoms (30–50%). Characteristic pulmonary findings encompass alveolar hemorrhage, interstitial lung disease (ILD), pulmonary infiltrates, and pleural effusion, while mediastinal adenopathy remains an exceptionally uncommon presentation of MPA [7,8].

Our case illustrates several remarkable features that underscore the diagnostic challenges inherent in atypical vasculitis presentations. The patient’s clinical picture was notably elusive, characterized exclusively by constitutional symptoms persisting for weeks without overt renal, neurological, or classic pulmonary signs. This atypical presentation, combined with his rural occupational exposure and serological findings suggesting *Coxiella burnetii* exposure, created a complex diagnostic scenario where infectious processes (particularly chronic Q fever) and neoplastic disorders represented the primary differential considerations. The recent 2022 ACR/EULAR classification criteria for MPA assign significant weight to MPO-ANCA positivity (+6) and ILD (+3) [1]. However, these criteria are specifically designed for application after histopathological confirmation of vasculitis and exclusion of mimicking conditions. Thus, our fundamental diagnostic challenge revolved around obtaining histological confirmation while navigating the complex differential diagnosis. The small size of the mediastinal nodes (9 mm) further complicated this endeavor, as conventional biopsy techniques were likely to yield insufficient material for definitive diagnosis.

In this challenging clinical context, EBUS-TMC emerged as a decisive diagnostic option. Using the Ariza-Pallarés four-step approach [4], the technique is embedded within linear EBUS to obtain sizeable, architecture-preserving cores from mediastinal or hilar lymph nodes. Pre-procedural planning begins with high-resolution chest CT (and PET–CT when available) to select the station, anticipate angulation or access hurdles, and avoid heavily calcified or necrotic targets; color Doppler mapping is performed upfront to delineate an avascular window and recognize arterial inflow patterns to avoid. The access point is chosen where airway mucosa and nodal capsule are thinnest and free of cartilage, ideally adjacent to the lesion. Under moderate sedation and standard monitoring, a two-operator setup (one managing scope/ultrasound, the other instruments) improves precision and safety. The sequence starts with a conventional EBUS-TBNA pass performed with Doppler activated to exclude intranodal vessels; several gentle, low-aspiration passes with a 22-gauge needle chiefly lay down an echogenic intranodal trail rather than seeking cytologic yield. Without changing scope angulation, a controlled transbronchial tunnel is then created by repeating TBNA without aspiration, shortening the stylet by ~1 cm to work across mucosa, submucosa, and capsule until the needle traverses the node smoothly, establishing a low-resistance path for the cryoprobe. A 1.1 mm flexible cryoprobe is advanced entirely under real-time ultrasound guidance; continuous B-mode tracking (with Doppler ready) maintains an avascular trajectory. To mitigate spatial heterogeneity, three cores per station (distal, medial, proximal) are typically obtained using a gentle fanning maneuver. Freeze cycles are brief (~3–5 s) to limit barotrauma and unintended tissue capture. After freezing, the bronchoscope–probe assembly is withdrawn en bloc; the core is released into saline and fixed in formalin. Each site is reassessed sonographically for bleeding, an occlusion balloon is kept available, and a post-procedure chest radiograph or pleural ultrasound is advised, followed by short observation and scheduled follow-up.

Operator experience is the main catalyst of EBUS-TMC performance: after ~30 supervised procedures, teams typically master three outcome-shifting skills—real-time selection of patients in whom preserved architecture will be decisive; Doppler-guided mapping of reproducibly avascular trajectories; and creation of a low-resistance tunnel with short freeze cycles (≈3–5 s) [9]. With this proficiency, nondiagnostic samples and crush artifact decrease, same-session definitive diagnoses increase, and rebiopsies decline. Within that framework, EBUS-TMC provides its greatest benefit in architecture-dependent diagnoses—especially lymphoma (where growth pattern and microenvironment matter), granulomatous disease (e.g., sarcoidosis or infectious granulomas, where distribution and necrosis guide interpretation), uncommon tumors, and benign conditions with patchy necrosis. The larger, minimally artifacted cores improve suitability for immunohistochemistry (IHC) and yield higher-quality DNA/RNA for extended panels, which is useful when broad ancillary testing is anticipated. By contrast, in metastatic carcinoma—particularly routine lung cancer staging—EBUS-TBNA often suffices: cytology usually establishes malignancy, and preserved architecture adds limited incremental value. In that setting, EBUS-TMC is reserved for a nondiagnostic TBNA, suspected concomitant lymphoproliferative or granulomatous pathology, or when additional tissue is explicitly required for biomarkers.

One of the earliest comparative clinical descriptions encapsulates the core advantage of EBUS-TMC in architecture-dependent diagnoses: in primary mediastinal seminoma, TBNA yielded crushed, non-diagnostic cells, whereas EBUS-TMC produced intact cores enabling definitive histology and IHC (e.g., SALL4, PLAP, D2-40, CD117), with only minor, self-limited bleeding and procedure time comparable to TBNA [10]. These mechanistic insights align with contemporary evidence showing that EBUS-TMC increases overall diagnostic yield, improves histo-architectural assessment, and provides higher-quality nucleic acids for ancillary testing relative to TBNA—thereby reducing the need for repeat procedures and enabling comprehensive molecular work-ups when needed. In a prospective series of patients with human immunodeficiency virus and mediastinal lymphadenopathy, EBUS-TMC achieved a high standalone yield and performed even better when combined with bronchoalveolar lavage (BAL), without observed complications—underscoring its value in benign and infectious conditions where needle cytology is frequently insufficient [11]. From the pathologist’s perspective, EBUS-TMC provides larger fragments, superior architectural assessment, and—in neoplasms—tumor cellularity >30% with substantially greater DNA/RNA amounts than TBNA, enabling comprehensive molecular testing and reducing rebiopsy rates with a favorable safety profile [12].

In a randomized study of 197 patients, transbronchial mediastinal EBUS-TMC outperformed TBNA in overall diagnostic yield (91.8% vs. 79.9%; *p* = 0.001). Yields were similar for metastatic lymphadenopathy (94.1% vs. 95.6%; *p* = 0.58), but EBUS-TMC was superior for uncommon tumors (91.7% vs. 25.0%; *p* = 0.001) and benign disorders (80.9% vs. 53.2%; *p* = 0.004). Starting with either technique did not materially affect final yield. Adverse events were infrequent (two pneumothoraces and one pneumomediastinum, all self-resolving; bleeding generally minor). EBUS-TMC cores were larger (mean diameter ~4.6 mm; area ~10.7 mm^2^) and, in non–small cell lung cancer (NSCLC), more often adequate for gene testing (93.3% vs. 73.5%), at the cost of a modest increase in sampling time (~11.7 vs. 9.4 min) [13].

In a systematic review by Botana-Rial et al. [3], prospective evidence comparing EBUS-TMC with EBUS-TBNA for intrathoracic lymphadenopathy was synthesized across seven studies (~555 patients). Despite technical heterogeneity (freeze 3–7 s; 1–3 cryo passes; with/without needle-knife), a consistent signal emerged: EBUS-TMC achieved a higher overall diagnostic yield (~92%) than EBUS-TBNA (~80%), with the largest incremental benefit in architecture-dependent conditions—notably lymphoma (diagnosis and subtyping), benign/granulomatous disorders (e.g., sarcoidosis, tuberculosis, pneumoconiosis), and uncommon tumors. In routine metastatic lung cancer staging, yields were broadly similar, supporting a selective step-up to EBUS-TMC after a nondiagnostic TBNA or when extensive ancillary testing is anticipated. Tissue adequacy favored EBUS-TMC: larger, minimally crushed cores more often supported comprehensive immunohistochemistry and molecular profiling (including PD-L1 and broad gene panels) than TBNA specimens. Safety was acceptable; minor airway bleeding predominated, while pneumothorax and pneumomediastinum were uncommon and self-limited, and no mediastinitis or procedure-related deaths were reported under standardized precautions. Practice implication: keep EBUS-TBNA as first-line for common metastatic indications, and escalate in the same session to EBUS-TMC when an architecture-dependent diagnosis is likely or cytology is inadequate—an approach that can reduce rebiopsies, shorten time-to-diagnosis, and secure tissue sufficiency for definitive histopathology and biomarkers.

In our case, EBUS-TMC was uneventful, with no bleeding, pneumothorax, pneumomediastinum, or infectious events. Contemporary series and pooled analyses report low complication frequencies when standardized steps are followed—short freeze times, Doppler-guided avascular trajectories, controlled tract creation, and planned hemostasis. Across studies, clinically significant bleeding is uncommon (~1%), while pneumothorax and pneumomediastinum are rare (<1%) and typically self-limited [14]. No cases of mediastinitis have been reported under these protocols. These data support a favorable risk–benefit profile in experienced hands and justify same-session escalation after a non-diagnostic TBNA in architecture-dependent scenarios.

The histopathological demonstration of neutrophilic small-vessel vasculitis with fibrinoid necrosis provided the definitive diagnostic evidence, but several converging factors supported the diagnosis of MPA while excluding alternative etiologies. The highly specific, high-titer MPO-ANCA (>134 U/mL), the presence of incipient pulmonary fibrosis on imaging, negative microbiological cultures, and the absence of clinical response to appropriate antibiotics collectively reinforced an autoimmune process. Infectious causes (particularly Q fever) were specifically reconsidered: the serologic pattern (IgG phase II ≥1:200 with phase I ≥1:50 and negative IgM) is more consistent with past exposure or chronic infection rather than acute disease; however, there were no clinical or imaging features of chronic Q fever (e.g., endocarditis or vascular infection), and echocardiography was unremarkable. Moreover, the patient failed to improve with azithromycin, levofloxacin, and doxycycline, while symptoms resolved only after immunosuppression, arguing against active infection. Finally, although *Coxiella burnetii* has been associated with secondary vasculitides (predominantly mixed cryoglobulinemic vasculitis), MPO-ANCA positivity has not been specifically documented in *C. burnetii*-related vasculitis, further supporting MPA over an infectious mimic [15,16].

To our knowledge, this represents the first reported case of MPA diagnosed via EBUS-TMC specifically targeting mediastinal adenopathy. While a single previous report describes EBUS-TMC for granulomatosis with polyangiitis affecting lung parenchyma [17], our case demonstrates the successful application of this technique for diagnosing MPA presenting with isolated mediastinal involvement. Because nodal small-vessel vasculitis is defined by vessel-centered injury (fibrinoid necrosis and leukocytoclasia) within specific nodal compartments, diagnosis relies on preserved architecture to identify and evaluate intact vessel walls—something that cytology-dominant TBNA rarely provides. Architecture-preserving EBUS-TMC cores therefore enable confident recognition of true vasculitic injury and exclusion of mimics. This extremely limited experience highlights the novel nature of our findings and suggests that EBUS-TMC for systemic vasculitides remains an emerging diagnostic approach rather than an established practice. Future research should explore several promising avenues. The comparative effectiveness of EBUS-TMC versus surgical approaches for vasculitis diagnosis warrants investigation through randomized trials. Additionally, the development of standardized protocols for processing and interpreting EBUS-TMC specimens in suspected vasculitis cases would enhance diagnostic reproducibility across institutions.

The primary strength of this report lies in its description of a novel application of minimally invasive technology to resolve a complex diagnostic dilemma, thereby preventing the need for more invasive surgical procedures such as thoracotomy or mediastinoscopy. To our knowledge, this represents the first reported case of MPA diagnosed via EBUS-TMC specifically targeting mediastinal adenopathy. The technique’s ability to provide adequate tissue samples from small mediastinal nodes while maintaining an excellent safety profile represents a significant advancement in diagnostic pulmonology. However, limitations must be acknowledged. The single-case design inherently limits generalizability, and larger prospective studies are needed to validate the utility of this approach for systemic vasculitides. Additionally, the diagnosis rests on tissue from a single mediastinal lymph node, introducing potential sampling bias for a systemic vasculitic process. Finally, no renal biopsy was performed—despite preserved renal function—so kidney histology is unavailable, which some MPA classification approaches consider for comprehensive confirmation; the proposed diagnostic algorithm, while successful, requires validation in larger cohorts to establish its sensitivity and specificity for vasculitis diagnosis.

## 5. Conclusions

In conclusion, EBUS-TMC represents a valuable diagnostic tool in the evaluation of unexplained mediastinal adenopathy, even when serological findings suggest alternative diagnoses. This technique proves particularly useful for diagnosing vasculitides with atypical presentations and may prevent unnecessary invasive procedures. As experience accumulates and technology advances, EBUS-TMC will likely assume an increasingly important role in the diagnostic algorithm of complex mediastinal pathologies, particularly for systemic disorders requiring histological confirmation. We propose a pragmatic pathway: start with EBUS-TBNA for most mediastinal nodes; escalate within the same session to EBUS-TMC when cytology is non-diagnostic or when an architecture-dependent diagnosis is likely (lymphoma, granulomatous disease, vasculitis). This approach maximizes first-pass adequacy while minimizing surgical procedures.

## Figures and Tables

**Table 1:** Clinical Timeline of Presentation, Diagnosis, Treatment, and 6-Month Follow-up.

Time Since Symptom Onset	Clinical Event	Diagnostic Findings/Interventions	Outcome/Response
Week 0	Symptom onset (fever, asthenia, weight loss)		
Weeks 1–4	Outpatient management	Empirical antibiotics (azithromycin, levofloxacin)	No clinical improvement
Week 5	Hospital admission	Initial lab workup: Leukocytosis, anemia, high CRP/ESR. Positive *C. pneumoniae* serology Positive p-ANCA/MPO.	Doxycycline initiated
Week 6	Ongoing multidisciplinary workup	Serology for *C. burnetii* positive (IgG). PET-CT: mediastinal adenopathy.	No response to doxycycline. Symptoms persist.
Week 7	Interventional procedure	EBUS-TMC of subcarinal lymph node performed.	Procedure well-tolerated. No complications.
Day 1 post-procedure	Histopathological results available	Definitive diagnosis: Microscopic polyangiitis (neutrophilic small-vessel vasculitis).	Immunosuppressive therapy initiated: Methylprednisolone (1 mg/kg/day) + Rituximab 1000 mg infusion (dose 1 of 2).
Day 3 post-procedure	Hospital follow-up		Complete resolution of constitutional symptoms. Hospital discharge
Day 15 post-procedure	Rheumatology outpatient follow-up.		Day-hospital treatment: Rituximab infusion Patient asymptomatic
6 months post-discharge	Rheumatology outpatient follow-up.	Negative p-ANCA. Normal CRP/ESR.	Rituximab maintenance (1000 mg × 2, 15 days apart). Patient asymptomatic

## Data Availability

The data underlying this article are available in the patient’s medical records at Complejo Asistencial Universitario de León. Anonymized data will be shared on reasonable request to the corresponding author.

## Abbreviations

The following abbreviations are used in this manuscript:

ACR/EULAR	American College of Rheumatology/European Alliance of Associations for Rheumatology
ANCA	anti-neutrophil cytoplasmic antibodies
CRP	C-reactive protein
CT	computed tomography
EBUS	endobronchial ultrasound
EBUS-TBNA	endobronchial ultrasound-guided transbronchial needle aspiration
EBUS-TMC	endobronchial ultrasound–guided transbronchial mediastinal cryobiopsy
ESR	erythrocyte sedimentation rate
H&E	hematoxylin and eosin
IgG	immunoglobulin G
IgM	immunoglobulin M
ILD	interstitial lung disease
MPA	microscopic polyangiitis
MPO-ANCA	myeloperoxidase anti-neutrophil cytoplasmic antibodies
p-ANCA	perinuclear anti-neutrophil cytoplasmic antibodies
PET-CT	positron emission tomography–computed tomography
SUV	standardized uptake value
